# Circulating Metabolomic Analysis following Cecal Ligation and Puncture in Young and Aged Mice Reveals Age-Associated Temporal Shifts in Nicotinamide and Histidine/Histamine Metabolic Pathways

**DOI:** 10.1155/2021/5534241

**Published:** 2021-09-02

**Authors:** Anthony Cyr, Lauryn Kohut, Lauran Chambers, Sladjana Stratimirovic, Brian Zuckerbraun

**Affiliations:** ^1^Department of Surgery, University of Pittsburgh, Pittsburgh, Pennsylvania, USA; ^2^Surgical Service Line, VA Pittsburgh Healthcare System, Pittsburgh, PA, USA

## Abstract

**Introduction:**

Aged individuals are at higher risk for morbidity and mortality following acute stressors than similarly stressed young people. Evaluation of age-associated metabolic changes could lead to the identification of specific therapeutic targets to improve outcomes from acute stressors, such as infections, in the elderly. We thus compared the plasma metabolomes of both young and old mice following cecal ligation and puncture (CLP), an accepted model of acute infection and stress.

**Methods:**

Young (9-17 wks) and aged (78-96 wks) male C57bl/6 mice were subjected to a retro-orbital bleed and two-week recovery prior to sham surgery (laparotomy alone) or CLP. Animals were sacrificed at 4 h, 8 h, or 12 h following intervention, and plasma was isolated from blood for subsequent analysis. Metabolomic analysis of samples were performed (Metabolon; Durham, NC).

**Results:**

Aged animals demonstrated greater intraprocedural mortality than young (30.2% vs. 17.4%, *χ*^2^*p* = 0.0004), confirming enhanced frailty. Principal component analysis and partial-least squares discriminant analysis of 566 metabolites demonstrated distinct metabolomic shifts following sham surgery or CLP in both young and aged animals. Identification of metabolites of interest using a consensus statistical approach revealed that both the histidine/histamine pathway and the nicotinamide pathway have significant age-associated alterations following CLP.

**Conclusions:**

The application of untargeted plasma metabolomics identified key pathways underpinning metabolomic responses to CLP in both young and aged animals. Ultimately, these data provide a robust foundation for future mechanistic studies that may assist in improving outcomes in frail patients in response to acute stressors such as infection, trauma, or surgery.

## 1. Introduction

The medical advances of the past century, including the identification of antibiotics and the discovery and implementation of vaccine programs, have led to a significant increase in life expectancy worldwide. In turn, the number of individuals aged >65 years is expected to surpass 1.5 billion by 2050 [1]. This vast segment of the population requires special attention from the medical community, both for diseases that are specifically associated with aging and for the altered physiologic responses to disease processes. Trauma, sepsis, or acute critical illness pose a particular diagnostic and therapeutic challenge, as these are associated with increased morbidity and mortality among the elderly [2–4]. Much of our understanding of these poor outcomes has come from statistical analysis of frailty scores in the elderly patients presenting with emergency conditions, and historical studies have been limited in scope [2–4]. More recently, a large-scale study among patients at the Veterans Affairs Hospital Network affirms that mortality is broadly increased across all surgical risk strata among the most frail patients, even in elective and optimized situations [5]. Collectively, these observations underscore the critical burden that an aging and increasingly frail populace pose to society—both for the patients themselves who experience higher risks and for the healthcare systems that provide complex care.

Aging itself is a progressive decline in functional capacity resulting from a combination of degenerative processes in cellular metabolism, with multiple consensus hallmark features [6–8]. DNA repair, epigenetic signaling, and protein homeostatic mechanisms become broadly deficient with aging across the phylogenetic landscape, leading to broad alterations in mitochondrial function and cellular bioenergetics. Modern high-throughput assays and systems biology tools have enabled the use of broad omics-based approaches to provide insight into these aging pathways [9]. In particular, metabolomic studies, which involve the identification of small (MW < 1500 Da) metabolites from isolated samples, have proven to have significant value [10]. As these small molecules represent the end result of layered epigenomic, transcriptomic, and proteomic regulation, metabolite profiles provide an integrative view of complex biological systems. In humans, studies utilizing metabolomic methods to help delineate age-associated patterns and patient subgroups in sepsis [11], pneumonia [12], and trauma [13] have proven to be of significant value in highlighting potentially novel avenues for both basic research and potential intervention. However, human studies remain costly and time-consuming, and murine models remain a mainstay for evaluation of central metabolic pathways following insult. Several groups have investigated the metabolomic signature of aging in mice [14–16]. Notable among these studies, Tomás-Loba et al. identified that metabolite signatures in aged mice correlated with biochemical aging as measured by telomere shortening rather than purely chronological aging [16], demonstrating the value of metabolomic profiling in examining the effects of aging on disease.

One of the key limitations of the murine analyses to date is the lack of evaluation of physiologic insult in young vs. aged animals. Based on prior murine metabolomic studies, we hypothesized that aging would be associated with distinct metabolomic trajectories following physiologic stress. To address this, and to bridge the gap between murine modeling and the human cohort data, we performed plasma metabolomic profiling of both young and aged mice at various time points following a standard cecal ligation and puncture (CLP) or sham surgical intervention. These data provide a robust signature both of the baseline metabolomic differences between young and aged mice and several differences in metabolite responses to surgical stressors and sepsis. In particular, we identified significant signature differences in the nicotinamide metabolite axis as well as in histidine and carnosine amino acid biology, which provide a clear path forward for future investigation and potential intervention to medically treat some of the biological sequelae of aging in the acute care setting.

## 2. Materials and Methods

### 2.1. Animal Studies

Animal protocols were approved by the University of Pittsburgh Institutional Animal Care and Use Committee. The experiments were performed in adherence to the US National Institutes of Health guidelines on the use of laboratory animals. C57BL/6 mice ages 11-18 weeks (young) or 86-96 weeks (aged) were utilized for the experimental protocol. For retro-orbital blood sample collection, intraperitoneal injection of a ketamine-xylosine mix was used for procedural sedation, and blood samples were harvested using heparinized capillary tubing. Animals were observed during immediate postprocedural recovery and then allowed to recover fully for a period of two weeks prior to randomization to either immediate sacrifice, sham surgical intervention consisting of a laparotomy and closure in layers, or cecal ligation and puncture done as previously described [17]. Antibiotics were not administered. At 0 h, 4 h, 8 h, and 12 h postprocedurally, animals were sacrificed by isoflurane asphyxiation and cervical dislocation, and blood was immediately collected *via* cardiac puncture for analysis. These timepoints were chosen based on data from biometric monitoring with implantable sensors following CLP, which underscored that initial physiologic changes take place within this time period following the initial CLP insult [17].

### 2.2. Sample Preparation

Following collection, all blood samples were stored briefly on ice in heparinized 1.5 mL microcentrifuge tubes. Samples were spun at 500 g for 15 minutes to isolate the plasma fraction, which was distributed into multiple aliquots of 150-200 *μ*L prior to flash-freezing in liquid nitrogen followed by long-term storage at -80°C.

### 2.3. Measurement of Plasma AST/ALT

Circulating AST/ALT was measured using the Liver Panel chemistry set and associated Heska analysis platform (Heska, CO, USA). Serial dilutions were performed of plasma samples to ensure appropriate reads within the linear range of the detector, as specified by manufacturer's instructions.

### 2.4. Untargeted Metabolomics

Plasma metabolomics was accomplished by Metabolon (Durham, NC, USA, http://www.metabolon.com) using a multiplatform system encompassing tandem ultrahigh-performance liquid chromatography-mass spectrometry as well as tandem gas chromatography-mass spectrometry systems with different column ionization parameters. Peak calling, compound identification, and batch-effect normalization accounting for day-to-day interrun variability for individual metabolites were accomplished according to Metabolon's proprietary methodology and internal library of standards. A total of 834 metabolites were identifiable, of which 566 had specific KEGG or HMDB identifier labels.

### 2.5. Metabolite Normalization and Statistical Evaluation

Statistical analysis as described in the text was undertaken using MetaboAnalyst (http://www.metaboanalyst.ca), an open-source suite of metabolomics analysis tools maintained by the Xia Lab at McGill University [18, 19]. Raw peak area data was imported, normalized to appropriate pooled control data, mean-centered, and scaled to the standard deviation of the metabolite across all samples prior to downstream statistical analysis, which included PCA, partial least-squares discriminant analysis (PLS-DA), empiric Bayesian analysis of microarray (EBAM), and random forest methods. Metabolites were rank ordered by importance by taking the median rank order of the results of these individual methods for downstream visualization purposes. The top 100 metabolites for individual comparisons as described in the text were subjected to pathway analysis within MetaboAnalyst, using the list of 566 input variables as a reference dataset to evaluate for pathway enrichment and statistical significance. Prism 8.4.2 (GraphPad Software, LLC, San Diego, CA, USA) was used to plot individual metabolite data. Two-way ANOVA was used to compare time trajectories of individual metabolites across different subgroups (young sham, young CLP, aged sham, and aged CLP). Post hoc multiple comparison testing was accomplished in Prism using Tukey's correction, and we specifically reported the comparisons between aged CLP and young CLP and aged sham and aged CLP.

## 3. Results

### 3.1. Aged Mice Are More Frail than Young

**Figure 1:**
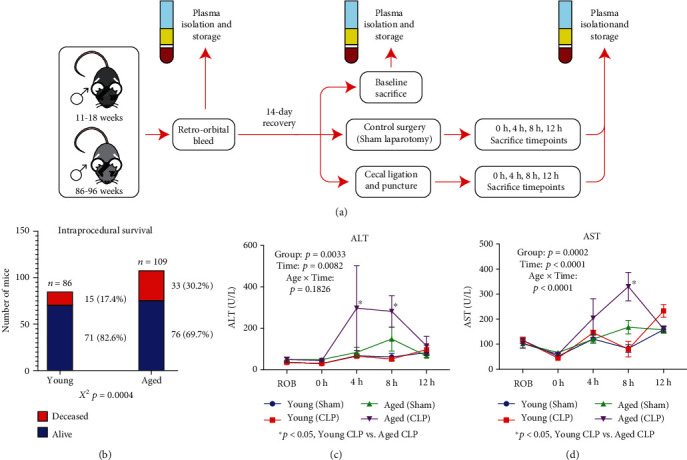
Summary of the experimental protocol and establishment of aged mouse frailty. (a) Graphical representation of the experimental design, including the sampling strategies and the various experimental groups included for analysis. (b) Description of the intraprocedural survival, demonstrating statistically significantly enhanced mortality for the aged animals in the protocol compared to the young. Alanine aminotransferase (c) and aspartate aminotransferase (d) levels in the plasma of animals at various timepoints, demonstrating increased levels of liver damage in the aged animals throughout the protocol when compared to the young animals.

Male C57BL/6 mice, aged either between 11 and 18 weeks (young) or 86 and 96 weeks (aged), were utilized for all studies according to the experimental protocol described graphically in Figure 1(a). To populate all experimental groups, a total of *n* = 86 young and *n* = 109 aged animals were required, with aged animals suffering statistically significantly elevated intraexperimental mortality, calculated as the number of animals who did not survive to the predetermined experimental timepoint (30.2% vs. 17.4%, *χ*^2^, *p* = 0.0004, Figure 1(b)). As an additional adjunct measure of frailty, we examined for liver injury using plasma alanine aminotransferase (ALT, Figure 1(c)) and aspartate aminotransferase (ALT, Figure 1(d)) using aliquots of plasma at the various isolated timepoints. For both ALT and AST, there were clear differences in ALT/AST trajectory over time, with aged animals demonstrating increased enzyme release in response to CLP compared to young animals. Taken together, these results suggest that aged animals have increased frailty to CLP compared to young animals.

### 3.2. Young and Aged Mice Have Distinct Metabolomes at Baseline

**Figure 2:**
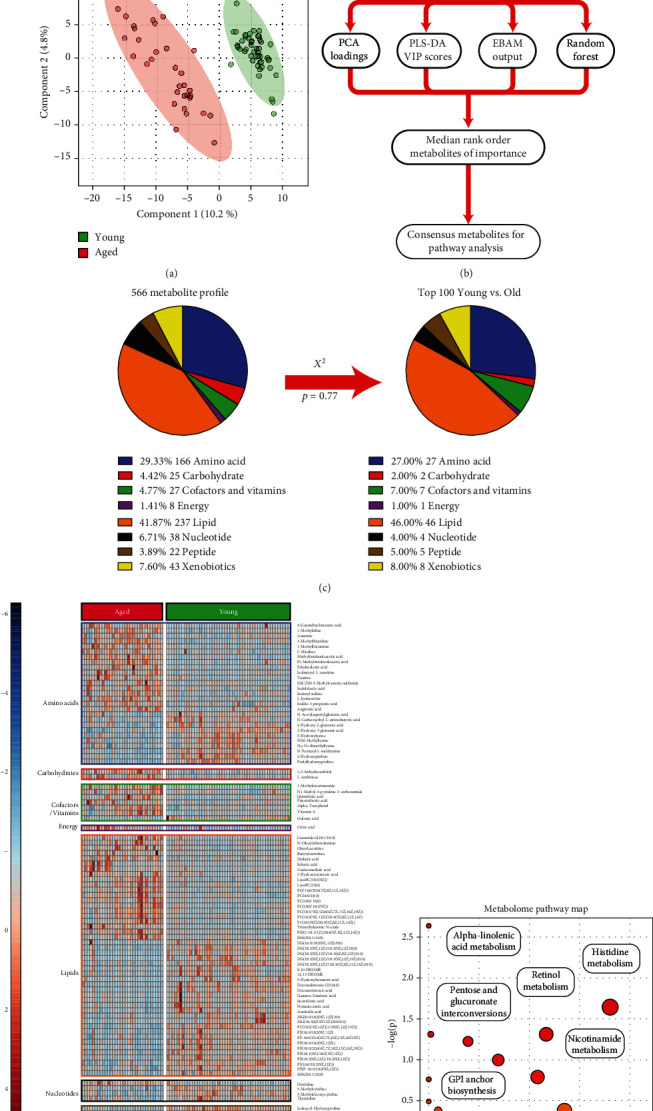
Comparison of baseline metabolomic status between young and aged animals. (a) PLS-DA plot of the metabolomic data from retro-orbital bleed samples from both aged (red) and young (green) animals, demonstrating distinct grouping and suggesting a clear difference at the metabolome level between young and aged animals at baseline. (b) Schematic representation of the statistical analysis algorithm used to identify the top 100 metabolites that best differentiate between young and aged animals at baseline. (c) Distribution of metabolite families among the original 566 metabolites in the dataset compared to the top 100 metabolites capable of differentiating young from aged. (d) Complete heatmap representing the top 100 metabolites, organized according to metabolite family. (e) Metabolome pathway map demonstrating specific metabolic pathway enrichment among the top 100 metabolites compared to the original 566 in the dataset.

Plasma samples isolated according to protocol were sent to Metabolon (Durham, NC, USA) for metabolite detection and annotation (see the Materials and Methods section for further details). A total of 834 discrete metabolites were identifiable across the dataset; 566 of which had identifiable Human Metabolome Database (HMDB) or Kyoto Encyclopedia of Genes and Genomes (KEGG, http://www.kegg.jp) accession numbers [20]. Raw metabolite peak areas provided by Metabolon were analyzed using the open source MetaboAnalyst package available at http://www.metaboanalyst.ca [18]. A flowchart illustrating data management strategies and representative output is in Figure S1. Initial attention was focused on identifying the major differentiating metabolites distinguishing the young from aged animals at baseline using the retro-orbital plasma isolates (Figure 2). Partial least squares discriminant analysis (PLS-DA) was used to visually identify broad metabolomic differences (Figure 2(a)), with clear delineation between aged and young animals. A consensus methodology was used to specifically stratify metabolites of interest; thereafter, integrating the contributions of principal component analysis (PCA), PLS-DA, empiric Bayesian analysis of microarray (EBAM), and random forest (RF) approaches to generate a consensus rank ordering of metabolites (Figure 2(b)). This did not produce a significant enrichment among any of the major categorical metabolite superfamilies (*χ*^2^, *p* = 0.77, Figure 2(c)). A detailed heatmap visualization of the top 100 metabolites, broken down by metabolite superfamily, is seen in Figure 2(d). As expected based on selection criteria, metabolites visualized in this fashion segregate into those that are predominantly enhanced in aged or young groups. To further break down key differences, we employed the pathway analysis algorithms available in MetaboAnalyst (Figure 2(e)). Here, we demonstrate that key differences between aged and young mice involve lipid biology (GPI anchor biosynthesis, *α*-linolenic acid metabolism), carbohydrate metabolism (pentose and glucuronate interconversions), cofactor and vitamin biology (nicotinamide and retinol metabolism), and amino acid regulatory pathways (histidine metabolism). Detailed information from this analysis is compiled in Figure S2.

### 3.3. CLP or Sham Surgery Induces Distinct Metabolomic Differences in Young Animals

**Figure 3:**
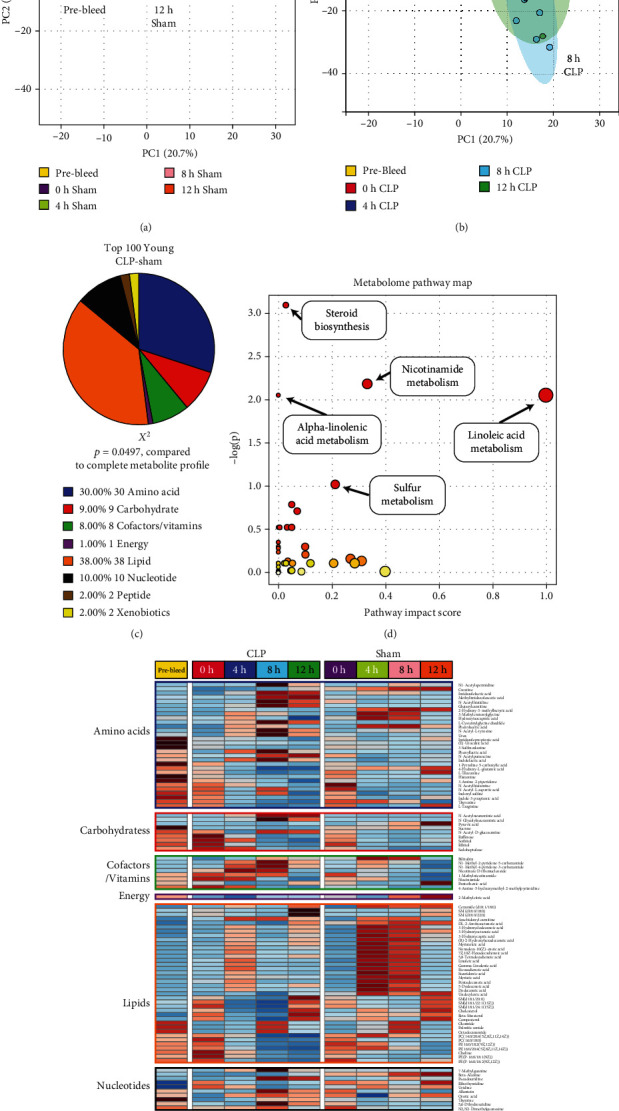
Identification of the metabolic pathways that drive the response to CLP in young animals. PCA plots of the young sham (a) and CLP (b) metabolomes. Sham-treated animals demonstrated a return to baseline over time, while CLP-treated animals did not. (c) Profile of the top 100 metabolites that differentiate between CLP and sham interventions in young mice over time. (d) Pathway analysis comparing enrichment of the top 100 metabolites compared to the 566 metabolites originally contained in the dataset. (e) Heatmap analysis demonstrating group average values of the top 100 metabolites at all CLP and sham surgical timepoints in young animals, illustrating broad differences in metabolomic responses following CLP compared to sham intervention.

We next isolated the metabolomic signatures associated with CLP or sham surgery specifically in the young population. For sham, the metabolomic response was to initially migrate from the retro-orbital bleed baseline (4 h and 8 h), but by 12 h, there was a notable colocalization with the retro-orbital bleed metabolome (Figure 3(a)). In contrast, CLP produced a persistent shift away from the baseline, with 4 h, 8 h, and 12 h timepoints all remaining distinct from the retro-orbital bleed samples (Figure 3(b)). Using the same consensus approach described above, we isolated the top 100 metabolites as features of interest. This generated a statistically significantly different distribution of metabolites within superfamilies compared to the original dataset (*χ*^2^, *p* = 0.05, Figure 3(c)). Key metabolite subpathways identified with pathway analysis tools included, as with the young vs. aged comparison, nicotinamide metabolism and *α*-linolenic acid metabolism. Pathways distinct to the young CLP-sham analysis included steroid biosynthesis, linoleic acid metabolism, and sulfur metabolism (Figure 3(d)). A detailed heatmap view demonstrates mild differences between CLP and sham among the young animals over different timepoints, particularly within the lipid superfamily, whereby a significant induction of certain lipid moieties dominates the 4 h-8 h sham timepoint prior to a return to retro-orbital bleed baseline values (Figure 3(e)).

### 3.4. CLP and Sham Induce Significant Metabolomic Changes in Aged Animals

**Figure 4:**
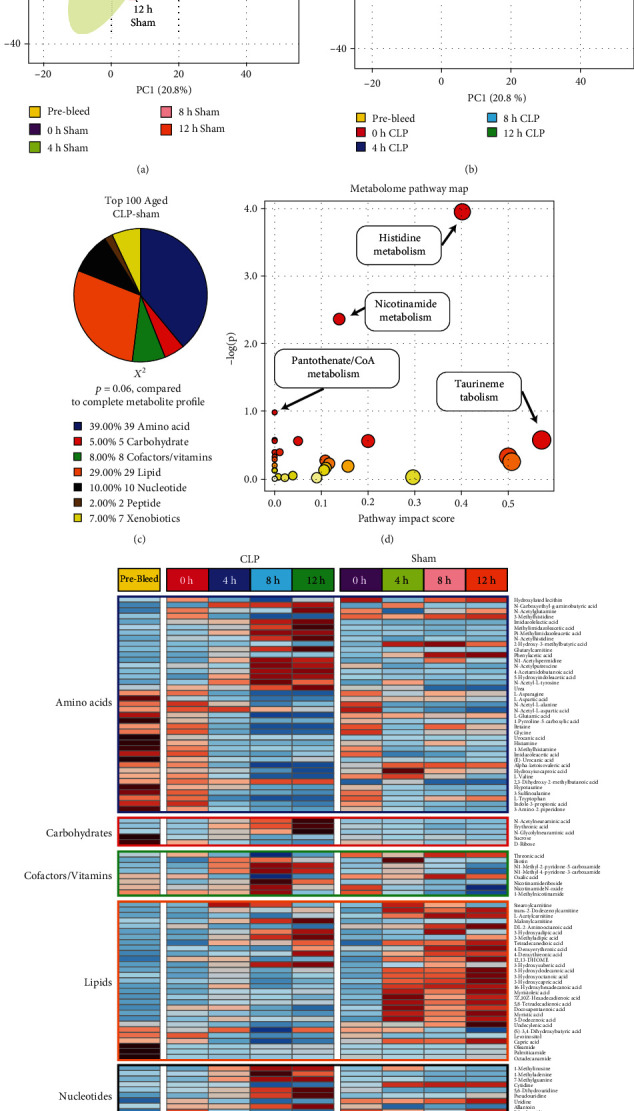
Identification of the metabolic pathways that drive the response to CLP in aged animals. PCA plots of the aged sham (a) and CLP (b) metabolomes. Sham-treated animals demonstrated a slow return to baseline over time, though not to the same degree seen in young animals. On the other hand, aged CLP-treated animals experienced progressive derangement of the metabolome over time. (c) Profile of the top 100 metabolites that differentiate between CLP and sham interventions in aged mice over time. (d) Pathway analysis comparing enrichment of the top 100 metabolites compared to the 566 metabolites originally contained in the dataset. (e) Heatmap analysis demonstrating group average values of the top 100 metabolites at all CLP and sham surgical timepoints in aged animals, illustrating broad differences in metabolomic responses following CLP compared to sham intervention.

Next, we examined the metabolomic effects of CLP or sham on the aged animal groups. In contrast to the young animals, sham intervention in the aged animals produced a differentiation from the retro-orbital bleed metabolome that persisted throughout the assayed duration (Figure 4(a)). Similarly, CLP induced large deviations away from retro-orbital bleed samples, with the magnitude increasing over the time course (Figure 4(b)). The pattern of 100 top metabolites isolated using consensus approaches was not statistically significantly different from the original metabolite pool (*χ*^2^, *p* = 0.06, Figure 4(c)). Significant metabolic pathways isolated in the aged population included histidine metabolism and nicotinamide metabolism, both of which overlap with analyses demonstrated above. Additional pathways unique to the aged population include pantothenate/CoA metabolism and taurine metabolism (Figure 4(d)). Heatmap evaluation of the average metabolomic responses across all groups for the top 100 consensus metabolites demonstrates more robust differences between CLP and sham surgery than seen in the young animals (Figure 4(e)). Notably, multiple amino acid derivatives and cofactors demonstrate significant enrichment at late CLP timepoints, whereas a sham intervention yields a more robust induction of key lipids at late timepoints.

### 3.5. Selection of Critical Metabolic Pathways for Further Evaluation

Having established key differences between young and aged animals at baseline, as well as critical pathways associated with the response to surgical stress in both young and aged animals, temporal trends within critical pathways across the entire dataset were evaluated. Tabulated outputs from MetaboAnalyst pathway analysis tools were compared, identifying overlapping metabolic pathways among the three comparator groups (Figure S2). Nicotinamide metabolism was the sole pathway represented among all three comparisons, while histidine metabolism was significantly enriched both for the retro-orbital bleed young versus aged comparison and the aged CLP-sham comparison. Notably, both *α*-linolenic acid metabolism and linoleic acid metabolism were identified in the young versus aged retro-orbital bleed group and the young CLP-sham comparison; however, on closer examination of these pathway structures, these were not informative and largely incompletely represented by the raw data (data not shown). Thus, detailed statistical analysis on both histidine metabolism and nicotinamide metabolism was performed.

### 3.6. Histidine Metabolism Critically Distinguishes Aged from Young Metabolomic Responses to Surgical Stress

**Figure 5:**
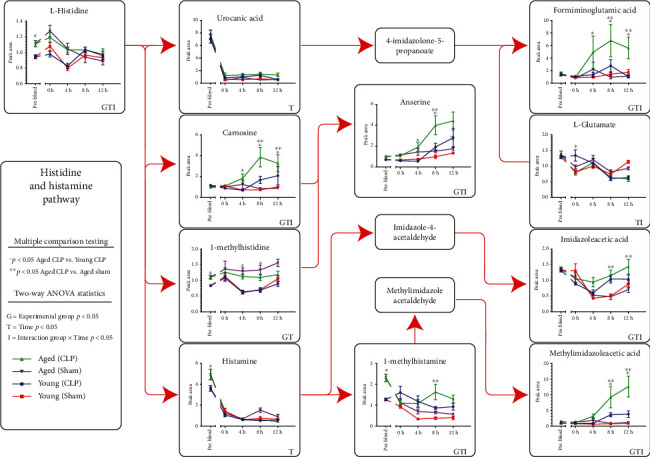
Detailed view of the histidine-histamine metabolic pathway. Schematic representation of the histidine-histamine pathway organized according to KEGG pathway diagrams, with individual values plotted for each of the four experimental groups (young sham, young CLP, aged sham, and aged CLP). Data was graphed for all available metabolites in the pathway, and undetected metabolites were left as labeled boxes. Statistical testing was accomplished using two-way ANOVA, and significance in this analysis was denoted in the right lower corner of each metabolite graph using G (group), T (timepoint), or I (interaction). Post hoc multiple comparisons testing was done to evaluate the differences between both aged CLP and aged sham as well as aged CLP and young CLP, as described in the methods.

Using the raw data, the elements of the histidine metabolism axis over the time course of the experiment grouped by age and surgical intervention was graphed. A total of 11 metabolites from the annotated KEGG pathway (http://www.kegg.jp) were represented in the dataset and are organized according to biochemical relationships in Figure 5. Statistical analysis was accomplished using two-way ANOVA, and the different types of statistical significance (by group [G], by time series [T], or by interaction of group and time [I]) are noted. In addition, individual comparisons focusing on specifically the differences between young and aged animals undergoing CLP, or differences between aged sham and CLP, were also accomplished. Arranging the data in this fashion demonstrated several clear patterns of metabolite trajectories. For some metabolites such as urocanic acid and histamine, CLP and sham intervention caused significant suppression without substantial variation in levels among groups. However, for many of the other metabolites, including carnosine, anserine, 1-methylhistamine, formiminoglutamic acid, imidazoleacetic acid, and methylimidazole acetic acid, a distinct increase was seen in the aged animals following CLP compared to either aged sham or young CLP, suggesting these metabolites could be age-associated markers of surgical sepsis. Still, other metabolites, including L-histidine and L-glutamate, had patterns that were less apparent, without clear distinction among groups at postsurgical timepoints.

### 3.7. The Circulating Nicotinamide Metabolome Is Significantly Altered following CLP in Aged Mice

**Figure 6:**
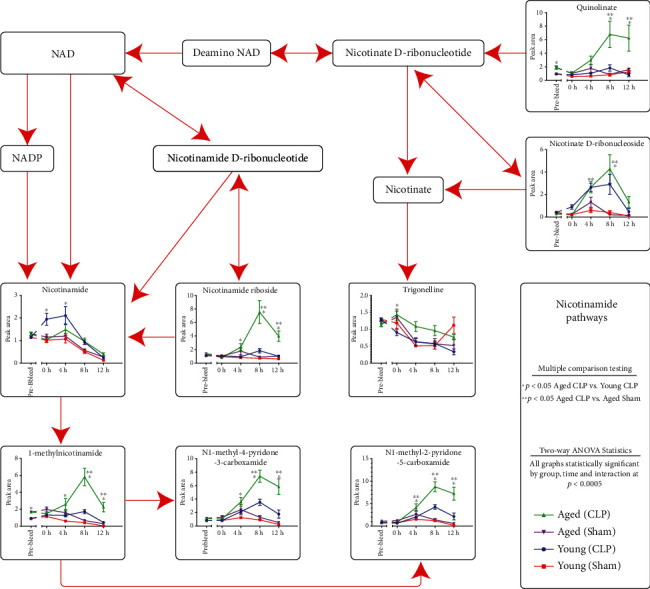
Detailed view of the nicotinate-nicotinamide metabolic pathway. Schematic representation of the nicotinate-nicotinamide pathway organized according to KEGG pathway diagrams, with individual values plotted for each of the four experimental groups (young sham, young CLP, aged sham, and aged CLP). As previously, data was graphed for all available metabolites in the pathway, and undetected metabolites were left as labeled boxes. Statistical testing was accomplished using two-way ANOVA, and significance in this analysis was denoted in the right lower corner of each metabolite graph using G (group), T (timepoint), or I (interaction). Post hoc multiple comparisons testing was done to evaluate the differences between both aged CLP and aged sham as well as aged CLP and young CLP, as described in the methods.

Based on the initial evaluation, individual metabolites in the nicotinamide metabolic pathway, as referenced from http://www.kegg.jp, were graphed (Figure 6). As above, these features were assessed both by two-way ANOVA and by individual multiple comparison testing approaches; notably, in this subset of data, all feature metabolites were significantly different both in terms of group comparison, time series, and interaction of group and time. Several key features are apparent on evaluation of the nicotinamide metabolite data. Of note, nicotinamide itself decreases over time, but has different early response patterns between young and aged animals, with the young animals demonstrating higher circulating levels of nicotinamide following CLP than sham or aged animals. However, for the majority of the downstream metabolites (nicotinamide riboside, 1-methylnicotinamide, N1-Methyl-4-pyridone-3-carboxamide, and N1-methyl-2-pyridone-5-carboxamide), the aged CLP animals had the most robust induction, compared to either the young CLP or the aged sham operations. Markedly, this pattern held for several detected nicotinamide precursor molecules, including nicotinate D-ribonucleoside and quinolinate. Taken together, these data demonstrate that there are significant differential responses to CLP between young and aged mice and suggest broadly that redox metabolism is altered in the aged response to surgical sepsis.

## 4. Discussion

Aging is a complex series of degenerative processes that results in the degradation of cellular homeostatic mechanisms and loss of metabolic, structural, and functional capacities that significantly impact the response to stress and illness. The data presented in this manuscript demonstrate clear baseline metabolomic differences between young and aged mice. These differences became more pronounced following surgical stress (sham operation) or surgical stress combined with infectious insult (CLP), underscoring the contribution of aging to metabolomic homeostasis. Furthermore, some metabolic pathways were found through statistical analysis to contribute more to the observed metabolomic alterations than others, highlighting important potential targets for the mitigation of age-associated adverse outcomes following surgical intervention.

The experimental design was chosen to provide maximal insight into multiple different metabolic trajectories. This is reflected in the specific analyses reported here, including initial comparison of young vs. aged animals, evaluation of global metabolomic trajectories from baseline in both young and aged animals individually, and in the final evaluation of pathways of overlapping significance between the young and aged animals. Critically, for the evaluation of each of these specific pathways, we utilized a consensus-based methodology combining multiple statistical analysis tools for the identification of key metabolites. This approach was selected to mitigate biases in individual feature identification approaches, and to limit the possibility that any specific methodology (RF, EBAM, PLS-DA, or PCA) would overfit the data. In particular, the use of the PCA loading scores among the data selection criteria provided an important counterweight to the supervised methods, as PCA loadings are agnostic to group assignment and are only sensitive to the total variance of the system. This allowed for the generation of a metabolite feature list incorporating both the broad structure of the data as well as key group-defining metabolites. This approach generated a robust profile of the broad metabolomic differences for each of the distinct experimental comparisons.

In the initial comparison, the baseline differences between young and aged animals from the retro-orbital bleed samples taken at the outset of the experimental protocol were evaluated. The data, summarized in Figure 2, demonstrates that no specific subclass among the top 100 metabolites significantly enriched above the distribution of the original dataset following the feature identification workflow (Figure 2(c)). As multiple means to identify metabolites based on group identification were used (PLS-DA, EBAM, RF), these 100 selected metabolites provided excellent discrimination between young and aged animals when displayed as a heatmap (Figure 2(d)). Notably, the findings here are concordant with many of the findings in prior studies, specifically relating to the identification of lipids and amino acids that strongly discriminate between aged and young mice [14, 16]. The pathway analysis also was concordant with prior studies, demonstrating significant enrichment of key antioxidant and redox pathways (nicotinamide metabolism, pentose interconversions) as well as lipid-specific signaling pathways (retinol, linolenic acid metabolism).

Additionally, these data examined the temporal changes in the metabolome following surgical sepsis (CLP) or sham operation in both young and aged animals. These data provide significant insight into some key differences underpinning differential responses to surgical stress in aged vs. young animals. A key difference is the broad metabolomic landscape shifts that occur in both young and aged animals and how these differ over time. PCA-based analysis and 2-dimensional plotting demonstrated distinct temporal patterns for sham surgical intervention and CLP in both age cohorts. Notably, sham surgery demonstrated a metabolomic shift away from both the retro-orbital bleed controls and the 0 h sacrifice, with an apparent movement back to baseline by the 12-hour timepoint for both young and aged animals (Figures 3(a) and 4(a)). Interestingly, this return to the 0 h/retro-orbital bleed baseline was more marked in the young animals, suggesting a more rapid recovery from sham surgery than seen in the aged animals. Collectively, these data underscore that the biological stress of surgical intervention is a significant insult to metabolomic equilibrium and is more pronounced with aging—a finding that has been routinely demonstrated in human cohorts. In contrast, the more pronounced stress of CLP induced a persistent metabolomic shift in both young and aged animals, with a more marked deviation from the control metabolome seen over time among the aged animals (Figures 3(a) and 4(b)). These data demonstrate clear metabolomic shifts following both sham surgery and CLP. For both the young and aged groups, statistically significant metabolite enrichment profiles were noted after application of the feature selection algorithms (Figures 3(c) and 4(c)), suggesting more narrow pathway alterations following these respective insults. Importantly, when evaluating these top 100 metabolites visually using heatmaps, some broad visual differences are apparent. Notably, in the sham surgery groups, the aged cohort appears to have persistent enrichment of several key lipids across time, whereas in the young sham intervention, there appears to be a transient enrichment followed by a return to a more “baseline” pattern (Figures 3(e) and 4(e)). This effect is less pronounced for the CLP groups, with substantial variation in the patterns identified therein. In the context of the broad metabolomic shifts identified on the 2D-PCA plots, this may be contributing to a delayed “injury recovery phase” for the sham surgical interventions in the aged mice compared to the young. Further studies with longer term timepoints are required to better characterize this trajectory.

In addition to the broad metabolome-wide trajectories identified among the groups noted above, key pathway enrichment results were examined for each of the main analyses (young vs. aged baseline, young CLP/sham, and aged CLP/sham) and overlapping pathways of interest were isolated across the three groups (Figure S2, Table S1). Within the histidine metabolism pathway, a key notable finding was the elevation of histidine derivatives carnosine and anserine predominantly in the aged CLP group, when compared either to aged sham or young CLP. Carnosine, a dipeptide comprise of *β*-alanine and L-histidine, is an bioactive molecule with pleiotrophic function in different tissue compartments, including antioxidant scavenging, pH buffering, metal chelation, inhibition of advanced glycation end product formation, and inhibition of advanced lipoxidation end product formation (Alexander A. [21]). These functions are integrated in several animal studies that have focused on its potential protective effects in ischemia-reperfusion injuries. This protective effect has been observed in the murine brain, liver, kidney, and testis and initially was suspected to be associated with the antioxidant capacity of carnosine [22–25]. However, it has been proposed that carnosine may also function via histaminergic signaling cascades as a reservoir for histdine/histamine, as several studies have demonstrated that subantioxidant doses of carnosine supplementation during renal ischemia-reperfusion injury modeling in both renal and cerebral perfusion were able to induce a renal protective effect [26]. In rat sepsis models, carnosine has also been shown to have protective effects both on liver function tests, markers of liver oxidative damage, and renal function [27–29]. Critically, carnosine also is suspected to have functions in modulating lifespan. Several groups have demonstrated that carnosine has variable effects on life span increases in murine models of accelerated aging [30, 31] as well as in cell senescence assays [32], though the specific mechanisms underpinning this are not fully elucidated yet. The present data, which demonstrate an enhanced circulating carnosine signature in aged mice with CLP (Figure 5), suggest an age-associated endogenous carnosine response to injury that merits further investigation for potential therapeutic leverage. To our knowledge, no current data links carnosine with the aged response to sepsis, which makes this a remarkable target for future study.

The other key pathway that emerged from statistical evaluation of the metabolite data involved nicotinamide biology (Figure 6). Here, the data was less complete, due to the inability of the utilized metabolomic platform to identify plasma NAD, NADP, or nicotinamide D-ribonucleotide, which are well-known key regulators of redox homeostasis and key drivers of oxidative metabolism. One hypothesis that this is due to the intracellular compartmentalization required to limit degradation of these labile redox species and suspect that any of these molecules in free circulation would be rapidly degraded or otherwise consumed by circulating redox enzymes. However, the detectable molecules in this pathway included several interesting species, including nicotinamide riboside, which is considered to be an orally bioavailable NAD precursor vitamin capable of increasing NAD levels in both humans and mice [33]. Critically, nicotinamide riboside has been shown in experimental models of murine sepsis to limit both pulmonary and cardiac toxicity, likely through NAD+/SIRT1 signaling, suggesting that this may be a key factor influencing outcomes in our CLP model [34]. Curiously, in the aged CLP animals, there was a statistically significant elevation in the circulating nicotinamide riboside compared to either aged sham or young CLP or sham animals. However, evaluation of the downstream metabolites in the pathway suggests that this spike is potentially not associated with an increase in NAD or NADP, but instead with enhanced degradation of NAD and its derivatives, as we see similar increases in both 1-methylnicotinamide and its metabolic products (Figure 6, bottom panels). Critically, these metabolites are known to be associated with signaling pathways involved in transitioning from carbohydrate to fat energy metabolism [35], as well as the pathophysiology of pulmonary artery hypertension in rat models [36]. Taken together, these findings suggest that standard supplementation of orally bioavailable NAD derivatives in the current model system may not improve functional outcomes, as nicotinamide riboside is already elevated. Further, they suggest that study of the downstream transformation products of NAD and its associated related metabolites may be playing a role in the aged murine response to CLP. While we hypothesize that this may be associated with increased tissue fragility and spillage of intracellularly quarantined NAD pools in the aged animals, these findings require further experimental investigation to determine specifically how the nicotinamide-NAD axis may be altered in the aging organism in sepsis and how it may be appropriately leveraged for clinical benefit. In particular, evaluation of the NAD salvage pathways, which have known implications in both lifespan and healthspan in various organisms, will be critical moving forward [37].

The work presented here, while providing intriguing evidence of circulating age-associated metabolic signatures in a murine CLP model, is not without limitations. The experimental design sought to develop a time course post-CLP that would capture a metabolic shift in the animals, based on telemetry data suggesting a biometric inflection point in the 5-10-hour range following CLP in young C57Bl/6 animals [17]. The PCA visualization of individual subgroups demonstrates that the postintervention timepoints (0 h, 4 h, 8 h, and 12 h) likely are adequate for capturing post-CLP metabolomic changes, but that more granularity would potentially be important for more specific biochemical pathway interrogation. This is particularly true given the increased intraprocedural mortality in the aged animal population, which suggests a more rapid deterioration following surgical intervention. This study was also initially conceived to be a one-to-one series with each animal providing its own control data. The goal of this was to provide data about individual metabolic trajectories, as opposed to comparisons of point measurements of the circulating metabolome, in young vs. aged animals following surgical intervention or septic insult. Unfortunately, due to unforseen logistical circumstances, it was not possible to progress with that initial design, but maintained the experimental protocol such that every animal entered into the experimental protocol underwent the same retro-orbital bleed prior to sham or CLP. Nonetheless, evaluation of these data as pooled populations remained robust. Finally, while the nicotinamide-NAD pathway remains an interesting target for future evaluation, this study also illustrates the limitations of the circulating metabolome for identification of labile redox-sensitive species. While this study provides compelling foundational evidence that the NAD axis is altered in aged animals following CLP, it is clearly not definitive in determining the mechanisms at play.

## 5. Conclusions

In summary, this manuscript presents a large-scale evaluation of the circulating plasma metabolome in both young and aged mice following CLP or sham surgical interventions. Several key metabolic pathways of interest were identified that differentiate between the young and aged response to surgical insult and subsequent infection providing significant foundational evidence for future study, including both mechanistic evaluation of the aged response to acute disease and potential therapeutic development.

## Data Availability

The data that support the findings of this study are available from the corresponding author upon request.

## Supplementary Materials

Supplementary Materials Table S1: summary of pathway analysis output from MetaboAnalyst utilized to identify pathway enrichment among the top 100 metabolites for each of the listed comparisons. Of these, the nicotinate and nicotinamide metabolism and histidine metabolism pathways were selected for downstream evaluation based on their presence in multiple comparisons as well as the number of represented metabolites in the pathway. Supplemental Figure S1: schematic of normalization and data processing workflow. (a) Workflow for data reduction from the 834 discrete initial metabolites to the 566 annotated metabolites, followed by the approaches used for data normalization and evaluation. (b) Graphical output from MetaboAnalyst demonstrating normalization approach. Supplemental Figure S2: Venn diagram of overlapping metabolic pathways among the three individual statistical comparisons. These included the young CLP/sham only, the aged CLP/sham only, and the young/aged baseline comparisons. Two pathways (histidine-histamine and nicotinate-nicotinamide) were chosen for further downstream evaluation based on the representative number of metabolites as well as their appearance in the metabolite features following statistical analysis.
