# Supplementary material for: Circulating Metabolomic Analysis following Cecal Ligation and Puncture in Young and Aged Mice Reveals Age-Associated Temporal Shifts in Nicotinamide and Histidine/Histamine Metabolic Pathways
Source: Oxid Med Cell Longev. 2021 Sep 2;2021:5534241. doi: 10.1155/2021/5534241 (PMC8433009; doi:10.1155/2021/5534241)
Supplement: Supplementary Materials — Table S1: summary of pathway analysis output from MetaboAnalyst utilized to identify pathway enrichment among the top 100 metabolites for each of the listed comparisons. Of these, the nicotinate and nicotinamide metabolism and histidine metabolism pathways were selected for downstream evaluation based on their presence in multiple comparisons as well as the number of represented metabolites in the pathway. Supplemental Figure S1: schematic of normalization and data processing workflow. (a) Workflow for data reduction from the 834 discrete initial metabolites to the 566 annotated metabolites, followed by the approaches used for data normalization and evaluation. (b) Graphical output from MetaboAnalyst demonstrating normalization approach. Supplemental Figure S2: Venn diagram of overlapping metabolic pathways among the three individual statistical comparisons. These included the young CLP/sham only, the aged CLP/sham only, and the young/aged baseline comparisons. Two pathways (histidine-histamine and nicotinate-nicotinamide) were chosen for further downstream evaluation based on the representative number of metabolites as well as their appearance in the metabolite features following statistical analysis. [file 5534241.f1.docx]

**Supplemental Materials**

**Table S1:**

|  | **Pathway** | **Total** | **Expected** | **Hits** | **Raw p** | **Impact** |
| --- | --- | --- | --- | --- | --- | --- |
| **Young Series Only** | Steroid biosynthesis | 3 | 1.066 | 3 | 0.043621 | 0.0282 |
|  | Nicotinate and nicotinamide metabolism | 8 | 2.8426 | 5 | 0.10767 | 0.33246 |
|  | alpha-Linolenic acid metabolism | 2 | 0.71066 | 2 | 0.12509 | 0 |
|  | Linoleic acid metabolism | 2 | 0.71066 | 2 | 0.12509 | 1 |
|  | Sulfur metabolism | 1 | 0.35533 | 1 | 0.35533 | 0.21277 |
|  | Amino sugar and nucleotide sugar metabolism | 4 | 1.4213 | 2 | 0.44677 | 0.07631 |
|  | Galactose metabolism | 7 | 2.4873 | 3 | 0.48072 | 0.07076 |
|  | **Pathway** | **Total** | **Expected** | **Hits** | **Raw p** | **Impact** |
| **Old Series Only** | Histidine metabolism | 12 | 4.0812 | 8 | 0.018081 | 0.40163 |
|  | Nicotinate and nicotinamide metabolism | 8 | 2.7208 | 5 | 0.090553 | 0.13816 |
|  | Pantothenate and CoA biosynthesis | 9 | 3.0609 | 4 | 0.36458 | 0 |
|  | **Pathway** | **Total** | **Expected** | **Hits** | **Raw p** | **Impact** |
| **Young vs Old Series** | alpha-Linolenic acid metabolism | 2 | 0.55838 | 2 | 0.076919 | 0 |
|  | Histidine metabolism | 12 | 3.3503 | 5 | 0.21745 | 0.36065 |
|  | Glycosylphosphatidylinositol (GPI)-anchor biosynthesis | 1 | 0.27919 | 1 | 0.27919 | 0.00399 |
|  | Pentose and glucuronate interconversions | 4 | 1.1168 | 2 | 0.31108 | 0.07812 |
|  | Nicotinate and nicotinamide metabolism | 8 | 2.2335 | 3 | 0.39488 | 0.13816 |
|  | Arachidonic acid metabolism | 2 | 0.55838 | 1 | 0.48146 | 0 |
|  | Linoleic acid metabolism | 2 | 0.55838 | 1 | 0.48146 | 0 |
|  | Glycerophospholipid metabolism | 9 | 2.5127 | 3 | 0.48233 | 0.21631 |

**Supplemental Figure S1**

**Supplemental Figure S2**
